# Parameters Influencing Cavitation Within Vials Subjected to Drop Shock

**DOI:** 10.1038/s41598-019-55668-9

**Published:** 2019-12-16

**Authors:** Rafael Valotta Rodrigues, Meagen Puryear, Donn Sederstrom, Corinne S. Lengsfeld

**Affiliations:** 1 University of Denver, Mechanical and Materials Engineering Department, Colorado, USA; 2 Johns Manville Company, Denver, Colorado, USA

**Keywords:** Mechanical engineering, Biomedical engineering

## Abstract

The pharmaceutical industry has made improvements to mitigate protein degradation during the drug manufacturing process, storage, and transportation. However, there is less quality control after the manufacturer releases the drug. Previous research has shown that drop shock due to mishandling and accidental dropping of therapeutic vials may cause cavitation, aggregation, and particle formation. In this study, the cavitation behavior of Deionized (DI) water and 10mM L-Histidine buffer solution which were subjected to drop shock by hand dropping were investigated to study the effects of vial materials, solutions, fill volumes, drop heights, and internal vial geometries. A Phantom v7 high-speed camera was used to record images at a rate of 66,700 frames per second of the vials as they underwent drop shock. These videos were then reviewed to find the angle of impact, and to determine if there was cavitation. The results indicate that decreasing fill height by using a smaller fill volume or larger diameter vials were found to mitigate cavitation across drop heights. Secondly, results indicate there is a significant difference between the cavitation behavior of glass and plastic vials, and plastic had more cavitation cases. Lastly, there was not a significant difference in the occurrence of cavitation between DI water and L-Histidine buffer solution.

## Introduction

Therapeutic proteins are used to treat many diseases, such as cancer, diabetes, hemophilia, Chron’s disease and rheumatoid arthritis^1^. These proteins are inherently unstable, which can cause aggregation and particles formation, ultimately leading to reduction of responsiveness to the drug therapy and eventually causing immunogenicity^1–9^. The pharmaceutical industry has made improvements to mitigate protein degradation during the drug manufacturing process, storage and transportation. However, there is less quality control after the drug is released by the manufacturer^4,5,9–12^. For instance, routine handling or unintentional mishandling of the drug may cause degradation to the drug but such factors are difficult or impossible to detect. There is a clear need to better understand risks associated with post-manufacturing handling of protein pharmaceuticals^4^. Research in this field should be addressed to verify if postproduction mishandling can cause protein degradation and to what extent, and to check if potential protein degradation byproducts interfere with safety and efficacy of the drug^4^.

Environmental stress factors typically found to happen in hospital or during self-administration by patients can influence protein stability. Potential issues with patients self-administered drugs include patient/caregiver intravenous administration, transportation of sub-cutaneous injection by patients to their homes, home storage which often deviates from recommended temperatures, involuntary agitation or drop of containers. Even exposure to light can be degrading for proteins, and containers are usually protected by a second container to avoid photodegradation. Regarding potential issues in hospital, protein stability can be compromised in hospital pharmacies if not properly and safely handled. Additionally, pneumatic tubes or hand carry to transport drugs from pharmacy to patients, not to mention that drugs are placed in temporary storage, can compromise protein stability. All those factors show that undoubtedly proteins must mechanically behave such that eventual mechanical stresses will not compromise its stability. Mechanical stresses are allegedly the main sources of stress that protein drug products are subjected to exposure during transportation processes and patients handling^4^.

**Figure 1:**
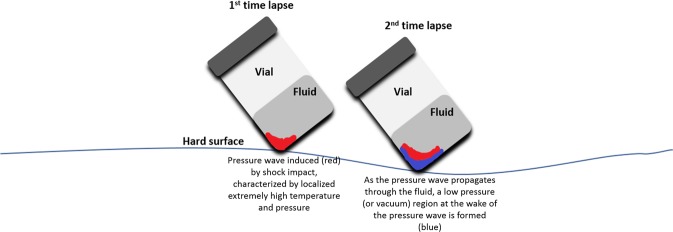
Pressure wave formed at the bottom of a vial that has been subjected to drop shock. The blue and red sections are representative of low- and high-pressure regions respectively.

Cavitation nuclei or spherical microbubbles are localized spots in which there is a phase change from liquid to vapor when the local pressure goes below the vapor pressure. These microbubbles can be located at walls or in the liquid. A phase change from liquid to vapor can happen when there is a sudden pressure drop at the fluid. An increase in viscosity can reduce the formation of bubbles. Transient isolated bubbles can occur at low pressure spots, disappearing at high pressure regions over the fluid flow. Cavitation typically occurs on wall geometries, which can induce increased local velocities, and pressure drops as a consequence^13^. Other examples of cavitation include cavitation induced by shock when a vial strikes a hard surface^5^, syringes under aspiration or ejection of fluid drugs^7,14^, and transportation of medical products using pneumatic systems^9^. Vials are one of the main modes of transportation for protein therapies and other drugs. During the process of shipping and administering these drugs, these vials can be accidentally dropped. When a vial is dropped cavitation can occur^5^. When a vial strikes a surface, there is a rapid deceleration which induces a high intensity pressure wave. A low-pressure region forms behind the pressure wave (Fig. 1) nucleating bubbles which subsequently collapse when ambient pressure returns. During the bubble collapse, some localized regions at the fluid are under extremely high intensity gradients of pressure and temperature, which induces the formation of a pressure wave that spreads throughout the fluid. These conditions are enough to degrade protein therapeutics^5,15,16^. Unless the vials shatter, the therapies are still administered to patients and can have adverse effects even if no visual changes have occurred^5^.

Regarding accidental drops, a study performed by Randolph *et al*.^5^ applied mechanical stress on vials filled with different antibody solutions. This study recorded micron-second video frames to physically characterize cavitation bubbles induced by a shock tower. Additionally, fluorescence spectroscopy determined if radicals were formed. Gelatinous particles were formed at the vials walls when they were under mechanical stress, and the number of particles at the wall increased as the height in which the vials were dropped increased. At a drop height of 40 inches, vials filled with 4 mL, 3 mL and 1 mL of Monoclonal Antibody 1 (mAb1) presented visible bubbles at the frame representing 500 μs, and they fully collapsed after approximately 1000 μs. Samples with DI water did not cavitate at this height. When the vial was oriented in a different way and dropped by hand from 1 m (100 cm) height, there was cavitation and the location of the bubbles varied according to vial orientation and the point in which the vial touched the surface. For the cases in which the angle of impact was 90° (vials in vertical position dropped using shock tower), multiple microbubbles oscillated after 30 μs and fully dissipated at approximately 120 μs. The behavior of the bubbles in the hand dropped cases in which there was an angle of impact different than 90° was the same, except that cavitation events lasted longer; 270 μs instead of 120 μs. The computational study based on a CFD (Computational Fluid Dynamics) model of the vial considered a drop height of 90 cm and three different vial materials: foam-padded 50 g, foam-padded 500 g, and metal. The pressure contours results showed low pressure regions at the bottom of the vials, demonstrating that hard impact metal cases are more likely to cavitate than the other two cases. The foam-padded materials represent the usual way in which pharmaceutical vials are packaged, while the metal material represents the behavior of vials without any packaging or protection.

Other examples in literature presented studies focusing on cavitation or vapor-liquid interfaces interaction, embracing pharmaceutical industry applications. For instance, Liu *et al*.^17^ investigated excipients and its effect on therapeutic protein aggregation. Ghazvini *et al*.^18^ developed a study to understand how particle formation and aggregation can be influenced by air-liquid interfaces under agitation, pH and solution. They found that aggregation is triggered at the interface by stress, but mechanical agitation played a large role on protein particle formation. Jayaraman *et al*.^19^ investigated protein aggregation caused by agitation, finding a close correlation between particles formation and fluid molecular characteristics, stability and hydrophobicity. Nayak *et al*.^20^ measured and compared subvisible particle formation for different filling technologies. This study used microflow imaging (looking at subvisible particles), size exclusion chromatography (looking at aggregates), and turbidity (looking at opalescence). The rotary piston pump showed a greater amount of subvisible particles in comparison with a rolling diaphragm, a peristaltic pump, and a time pressure filler. Bee *et al*.^21^ characterized aggregation under the application of shear rates on previously aggregated protein (mAb formulations). They concluded that micro-bubbles, adsorption, subvisible particles and stress related to pumping may play a larger role as sources of aggregation as compared to shear during production. Duerkop *et al*.^22^ experimentally and computationally (CFD) investigated if hydroxyl radicals can cause aggregation. They concluded that cavitation can cause aggregation, and aggregation due to cavitation is correlated to how stable the bubbles are and the quantity of interfaces. Duerkop *et al*.^23^ carried out a study to characterize mechanisms that can cause cavitation. By using a piston pump, they found that shear stress by itself did not alter protein structural properties. Torisu *et al*.^9^ investigated the effects of different vial fill volumes for an immunoglobulin G1 (IgG1) solution under two stress cases; small fill volume highly agitated and dropped from low heights, and a larger fill volume slightly agitated and dropped from higher heights. They found that the smaller fill volume resulted in a decrease in monomer area, as well as the lowest particle count. Additionally, Torisu *et al*.^9^ identified higher occurrence of aggregation when there is less headspace. While Kiese *et al*.^24^ may not have investigated cavitation phenomena, their research into characterizing the aggregation behavior of IgG1 for two mechanical stress methods (shaking and stirring) at two temperatures and varying amounts of polysorbate 20 (PS20) is crucial in understanding the effect that various fill volumes may have. A combination of shaking (without PS20) and head space resulted in aggregation. Whereas stirring induced visible particles, increased turbidity and subvisible particles but did not have an impact on soluble aggregates. A possible explanation is the rapid renewal of the air-solution interface during shaking, which may lead to adsorption and protein partially unfolding, and may also account for the increased particle counts for samples with more headspace during shaking.

All these studies mentioned above made excellent contributions to a better understanding of the mechanisms leading to cavitation in pharmaceutical industry. They prove that mechanisms leading to cavitation during drug manufacturing have been well characterized in literature. However, there is still a gap on pharmaceutical production chain after manufacturing, when patients and hospital pharmacies handle the drugs. Specifically, one of the crucial needs for pharmaceutical industry is the correct understanding of cavitation caused by accidental containers drop during patients handling or hospital pharmacies. The previous efforts carried out by Randolph *et al*.^5^ support the idea that cavitation in drug therapeutic vials is a real issue in pharmaceutical industry, but the literature review of this manuscript suggests that there is still a gap on understanding the mechanisms leading to this problem. While it is known that cavitation can accidentally be induced by shock in therapeutic vials during shipping and handling, there is need for an in-depth study to fully characterize aspects that mostly influence cavitation, and more importantly there is urgent need for developing mitigation strategies. This work aims to explore differences generated by change in vial materials, solutions, fill volumes, and internal vial geometries. By doing a full experimental characterization of cavitation occurrence, this study aims to establish a threshold for good practices on cavitation mitigation strategies for therapeutic drug vials.

## Methods

### Experimental overview

**Figure 2:**
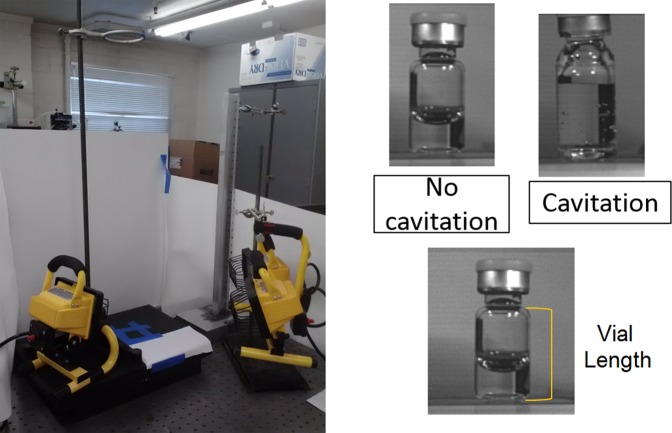
Experimental setup showing the apparatus used to investigate cavitation.

We developed an experimental approach to investigate cavitation on vials with different geometries during accidental drops. The experimental approach allowed not only to determine the effect of different drop heights on the onset of cavitation, but also to check how vial geometry influences bubbles formation. To simulate shock that could occur because of a vial being dropped unprotected onto a hard surface, numerous vials filled with a Deionized (DI) water and a buffer solution were hand dropped from varying heights onto a granite surface (Fig. 2). A high-speed camera was used to record the vials as they underwent drop shock. The presence of cavitation was then confirmed if the videos showed the creation of bubbles after impact.

### Vial information and sample preparation

DI water and a buffer solution were used for the study. The buffer solution was 10mM L-histidine at a pH of 5.0 filtered to 0.2 mm. Prior to sample placement and cleaning, the vials, plugs, and stoppers were weighed individually so that an accurate total mass could be calculated. The vials and their components were then placed inside the ultrasonic bath (*E/MC Corp*. *RAI Research*) with clean DI water and a small amount of soap (~1 drop) for three minutes. The ultrasonic bath was then emptied of the soapy water, so that the vial and its components could be rinsed with fresh DI water. Upon rinsing completion, the ultrasonic bath was again emptied and refilled so that the vials could sit in clean water. They were then removed from the ultrasonic bath and were airdried overnight upside down to mitigate any dust or other contaminants from entering the vials. Once dry, the insides of the vials were then wiped with Kimwipes (Kimberly Clark) to ensure that there were no remaining water droplets. This process was repeated until all vials and components were clean. The vials (West Inc.) used were made of two different materials; plastic and glass, as well as two different volumes; 2 ml and 5 ml. This resulted in five different glass vial variants (three 2 ml and two 5 ml vials) and three plastic vial variants (one 2 ml and two 5 ml).

**Figure 3:**
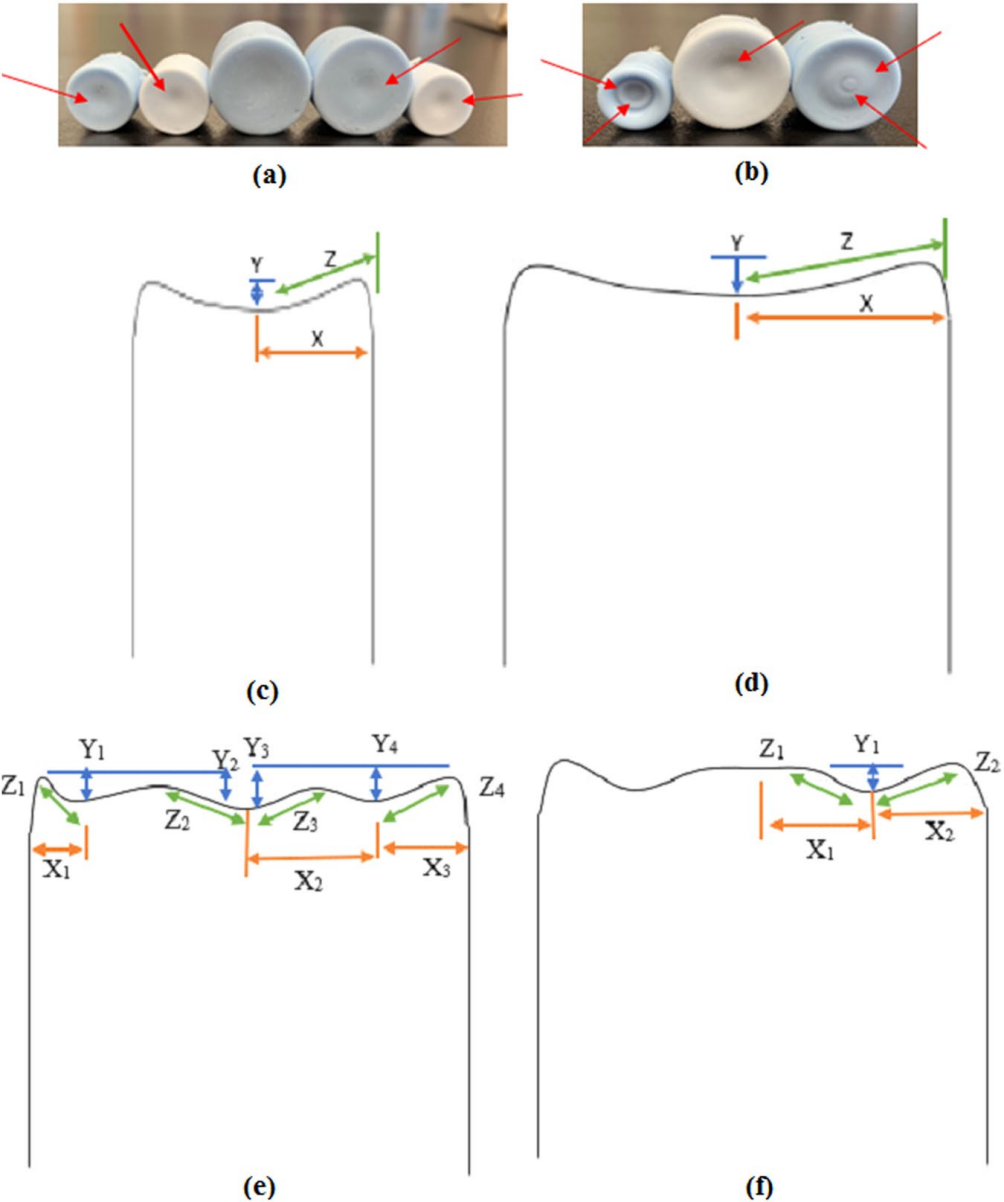
Molds of vial interior showing (**a**,**b**) the bottom geometry of the molds with inflection points for glass and plastic vials, respectively. Drawings of the mold bottoms showing dimensions of the (**c**) non-complex bottom geometry 2 mL vials and (**d**) non-complex bottom geometry 5 mL plastic vial A; (**e**) complex bottom geometry 2 mL plastic vial #1; (**f**) complex bottom geometry 5 mL plastic vial B.

Prior to being filled and dropped, the vial and its components (metal cap, rubber stopper, and plastic cap) were weighed individually, and both the vial body length and external base diameter was measured. Once all the vials were weighed, each different type (material and volume) were then averaged to have one specific weight for each type. The length of every vial body was measured from the base to the neck, and the external base diameter were measured using dial caliper and recorded (Fig. 2). Additionally, after vials were cut in preparation for casting molds the wall thicknesses of the glass vials were measured and recorded (Table 1). All vials were filled using a micropipette at ambient pressure and were sealed using industry standard crimping techniques. The 2 ml vials were filled with solution volumes of 1 and 2 ml. The 5 ml vials were filled with solution volumes of 1, 2, 3 and 4 ml. Each plastic vial had a minimum of two separate vial preparations, resulting in vials A and B (Fig. 3d,f, respectively).

**Table 1:** Vial wall thickness.

Type of Vial	Vial Wall Thickness (mm)
2 mL Glass Vial #1	1.11
2 mL Glass Vial #2	1.16
5 mL Glass Vial #3	1.33
5 mL Glass Vial #4	1.3
2 mL Glass Vial #5	1.16
2 mL Plastic	0.80
5 mL Plastic	0.80

The plastic vials are made from Poly(norbornene) - Cyclic Olefin Polymer (COP), and they have an O_2_ permeability of 1.2, a water vapor transmission rate of <10, a Youngs Modulus of approximately 2.6–3.6 GPa and a contact angle of 91 ± 2° (Stefaniu *et al*.^25^). The glass used is assumed to be the pharmaceutical standard of Type 1 borosilicate which is impervious to both O_2_ and water, a Youngs Modulus of approximately 63 GPa, and has a contact angle ranging from 5° to 60° depending on cleanliness (Personal Communication, *West Pharmaceutic Services* 2017).

### Fluid properties

In order to determine the fluid properties of the 10mM L-Histidine buffer solution, experimental data compiled (Table 2) by Ştefaniu *et al*.^25^ were used where they had looked at different molal concentrations of L-Histidine and L-Alanine in a NaCl solution of varying concentration. We specifically looked at the data for solution containing 0 mol kg^−1^ NaCl, meaning the solvent was bi-distillated water.

**Table 2:** Fluid properties of L-Histidine in bi-distillated water solutions at 298.15 K.

Amino acid molality (mol.kg^−1^)	ρ (kg m^−3^)	η (mPa s)
0.0000	997.04	0.8910
0.0511	999.93	0.8855
0.1003	1002.61	0.8996
0.1985	1007.88	0.9394
0.3021	1013.31	0.9802

Where ρ is the density of the solution, and η is the dynamic viscosity. Adapted from Ştefaniu *et al*.^25^.

Previously to using Ştefaniu *et al*.^25^ table for interpolation, conversions from molality to molarity had to be performed to determine if there was a significant difference between molarity and molality; the values used for the conversion were for a molality of 0.0511 mol kg^−1^. This was achieved by first assuming that there was 1 kg of solvent (water). Next, the total mass of the solute (L-Histidine) was found by multiplying the molality (0.0511 mol kg^−1^) of the solvent by its molar mass (155.1546 g mol^−1^). Then the total mass of the solution was found by adding the assumed solvent mass (1 kg) to the solute mass (7.928 g). The solution mass was then multiplied by the density (999.93 kg m^−3^) and divided by 1000 to find the solution volume (1.00785 L). Lastly, the molality was divided by the solution volume to give the molarity of 0.0507 mol L^−1^. In summary, the molality concentrations used by Ştefaniu *et al*.^25^ were small enough that molality and molarity were proved to be interchangeable. Thus, our concentration of 10*10^−3^ mol L^−1^ can be assumed to be equivalent to 10*10^−3^ mol kg^−1^ and used to interpolate density (ρ) and dynamic viscosity (η) values.

### Drop shock method

Preceding to videotaping the experimental drops, test drops were performed to ensure that an ideal impact target area (Fig. 2) was found and that the high-speed camera placed in an ideal location. The drop ring was used to ensure that each drop had the highest probability of impact in the target area. The granite slab was used as the impact surface due to the workbench having an uneven surface, which would make it hard to ensure repeatability. Once the ideal locations of both the impact target area and camera were found, the lens zoom, and camera settings were tweaked, along with lighting, to determine the optimum settings for the clearest videos. Over 230 vial drops were conducted using the five vial types. The vials were dropped one at a time by hand at heights of 20, 30, 40, 60, 80 and 100 cm. Each vial was dropped more than three times over several days so that the repeatability of the response could be quantified. If a vial had shattered upon impact, the granite surface was cleaned of all glass particles to avoid any unwanted interactions.

### Mold preparation and dimensions

Molds of the different vial types were created to observe the internal surface geometry of the bottom of the vials. This was done by using a diamond band saw to cut below the vial necks. The cut surfaces were then sanded to remove the rough edges for easier mold removal. The mold itself was silicon (Silicones, Inc). The silicon and activator were mixed with a ratio of ten to one (10:1) and was then put into a needleless syringe and squeezed into the precut vials. Once the silicon was dry the molds were removed; however, a release agent would be necessary to facilitate the ease of mold removal as they came out in pieces. Three potential release agents were used; spray canola oil, Rain-X glass water repellent, Vaseline and Ease Release 200 spray (Mann Release Technologies). The release spray was found to be the best release agent, followed by Vaseline. After creation and removal of the molds, it was found that the glass vials had flat bottoms (Fig. 3a), whereas the plastic vials had complex bottom geometries that consisted of varying inflections (Fig. 3b). After the molds were removed, measurements were taken of any present inflection points using a Mitutoyo dial caliper. The measurements included the depth (Y) of any inflection points and the horizontal distance (X) from the outer edge of the mold to the deepest part of an inflection point (Fig. 3c,d). If there were multiple inflection points, each of the vertical distances (Y_i_) were measured relative to the local highest point. In addition to the horizontal distance being measured from the outer edge of the mold to the deepest part of first inflection point, all subsequent horizontal distances (X_i_) were measured as the distance between inflection points (Fig. 3e,f). Once all horizontal and vertical distances were measured, the Pythagorean Theorem was used to calculate the slope (Z) of the inflection points (Table SM1 and Table SM2, Supplementary Material).

### High speed video

**Figure 4:**
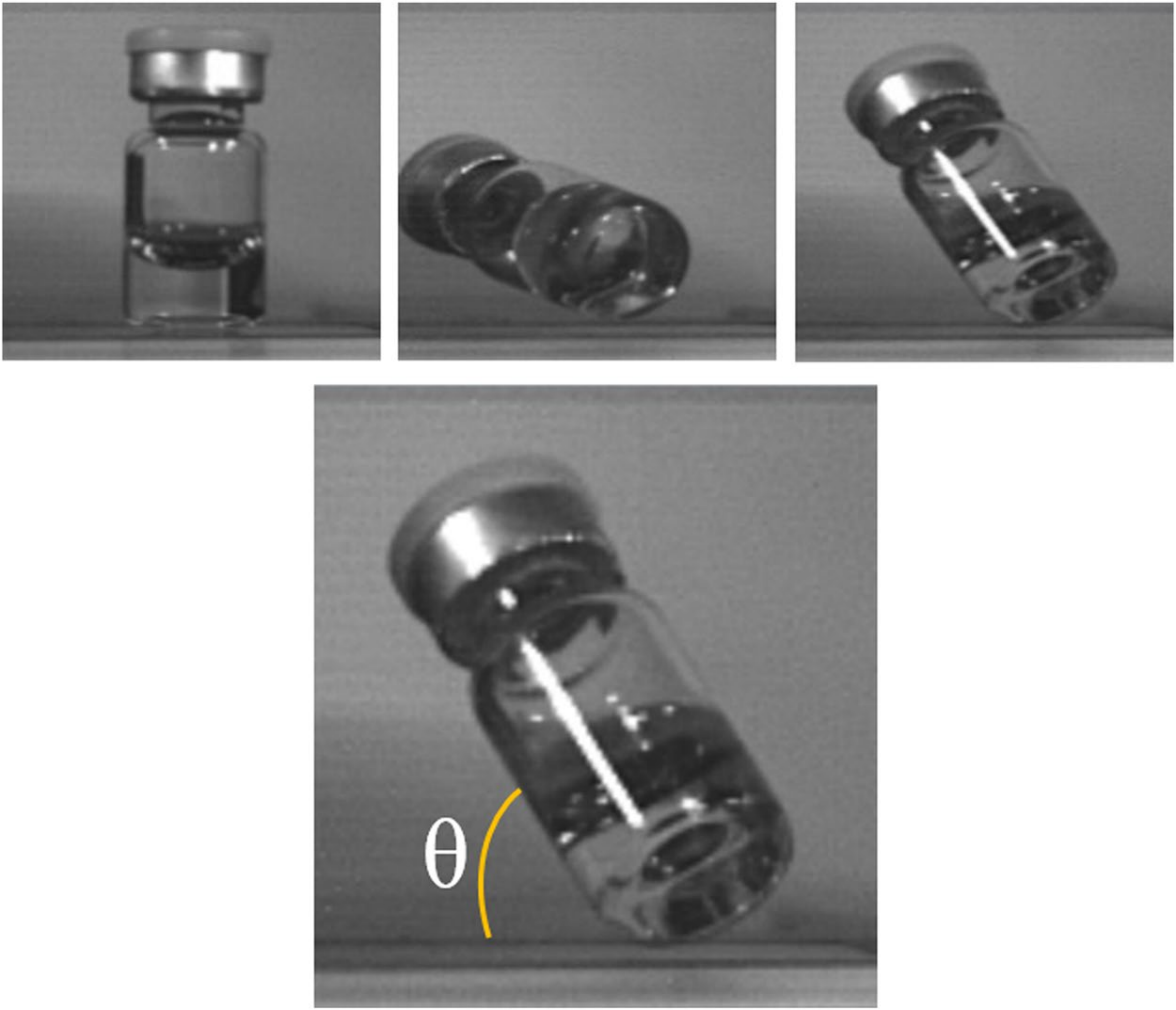
Angle of impact, showing visual representation of upright, side and 45–60 impacts respectively.

A Phantom v7 high speed camera was used to record images at a rate of 66,700 frames per second, with a resolution of 125 × 125 pixels, of the vials as they underwent drop shock. The camera had a DG MACRO 150 mm 1:2.8 lens. These videos were then reviewed to find the angle of impact (Figs. 2 and 4), height of the vial cap at impact, and to determine if there was cavitation. The angle of impact was measured by using the vial length, and the video software (CV 2.5, METEK) tools to calculate the sine of the drop angle (Fig. 4). The drop angles were divided into the following categories: side impact (<30°), 30–45°, 45–60°, 60–75°, and upright (>75°) (Fig. 4). The height of the vial cap at impact was found by measuring from the cap to the drop surface in the video software.

If cavitation occurred, the videos were further analyzed for: number of cavitation bubbles, if the bubbles oscillate, the length of time it took after impact for the onset of cavitation, and how long cavitation phenomena occurred. The number of cavitation bubbles that occurred were counted by pausing the video at the peak of cavitation. They were then grouped into <5, 5–10, or >10 bubbles present. If the bubbles faded in and out of the video, then it was determined that the bubbles oscillated. The videos were slowed down to 1fps to determine the onset of cavitation after impact, and the duration cavitation bubbles were present.

## Results

### Cavitation behavior: visual inspection

**Figure 5:**
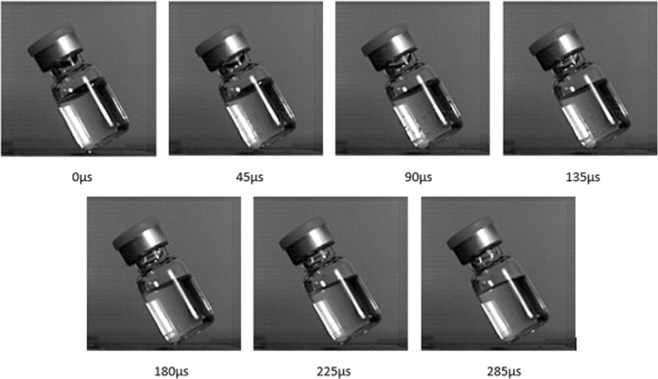
Cavitation time-lapse where bubbles flash across the entire fluid volume.

All drops were recorded, and the videos were then reviewed to determine if cavitation occurred. Figure 5 is an example of a vial with cavitation (video recording available as Supplementary Material – Video). In this example (Fig. 5), the onset of cavitation started 45 µs after impact with a few bubbles beginning to form, and bubbles becoming fully formed at 90–135 µs after impact. At 135–180 µs cavitation bubble formation began to decay but there were still a few bubbles at 180 µs. The cavitation process came to a completion at 225 µs, with all the bubbles having dissipated. At 60–80% of the total cavitation duration, corresponding to a time range of 135 µs to 180 µs, the largest bubble dissipation occurred. In comparison with the other cavitation cases, there was no clearly defined standard linking time lapses in which the cavitation process started or came to a completion.

### Angle of impact

**Figure 6:**
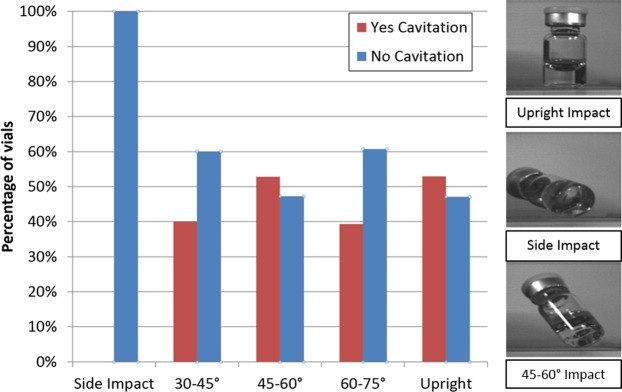
Percentage of cavitating and non cavitating vials based upon their impact angles.

Vials impacting between 30° and upright impact (>75°) were observed to have no significant difference between the occurrence of cavitating or non-cavitating drops. However, vials that impacted with a side impact (less than 30°) were observed to have no cavitation (Fig. 6). This is because the side impacts have a reduction in impact energy per impact area, under which conditions a pressure region with pressures below the vapor pressure do not occur. In other words, vials with side impacts will have the impact distributed throughout a larger surface in comparison with vials that impact the floor at a single point/location. Drops with an impact angle larger than 30° are subject to a low pressure region (below the fluid vapor pressure) at the bottom of the vials/near the impact area, the magnitude of which is dependent on the drop and impact conditions. Vials that impacted at angles larger than 30° experience a reduction in impact area. This reduction in turn causes the impact energy to be more concentrated in the fluid as compared to a side impact, where the side impact energy is more evenly distributed throughout the fluid/vial. The concentrated impact energy in the fluid of vials with an impact greater than 30° results in more cavitation.

### Fluid height and base area

**Figure 7:**
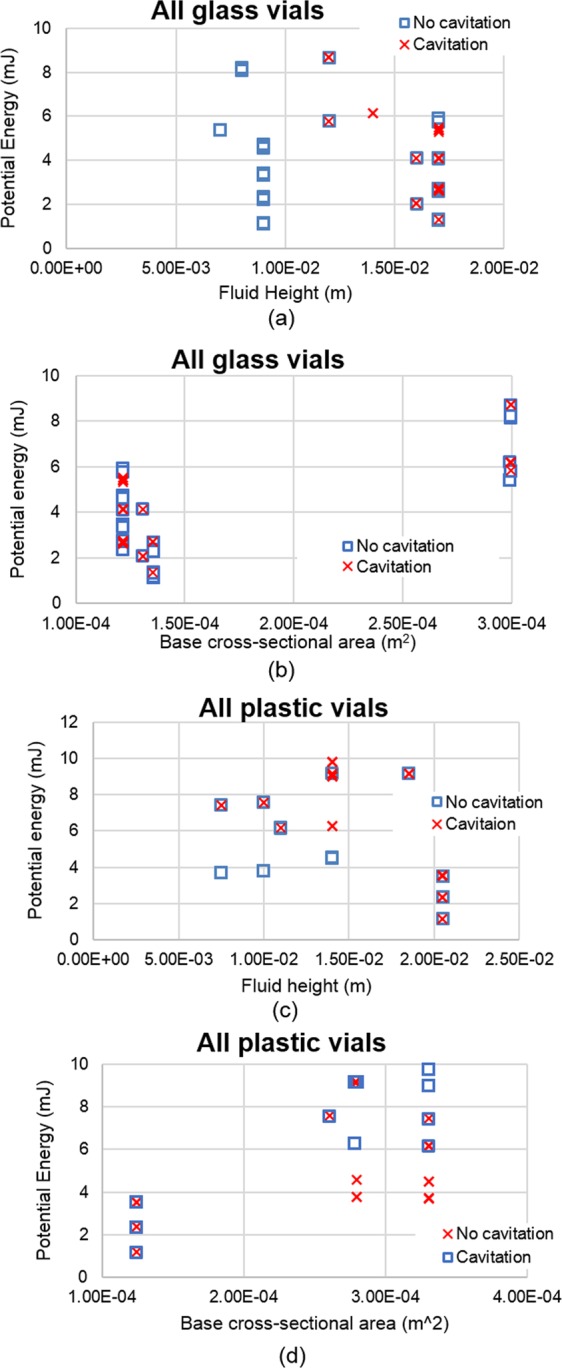
Potential energy (mJ). (**a)** Per fluid height for glass vials; (**b**) per base cross-sectional area for glass vials; (**c**) per fluid height for plastic vials; (**d**) per base cross-sectional area for plastic vials.

**Figure 8:**
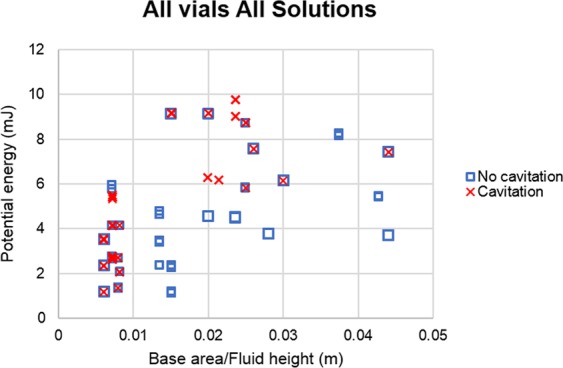
Base area/fluid height vs potential energy for all vials.

For glass vials, no cavitation occurred for vials with a fluid height under 0.01 m, no matter the drop height (Fig. 7a). Conversely, if the fluid height was greater than 0.01 m, cavitation occurred at all drop heights. The base cross-sectional area vs potential energy graph (Fig. 7b) does not provide a clear standard to distinguish the onset of cavitation. The factor driving cavitation in glass vials appears to be the fluid height. For plastic vials, no cavitation occurred at a fluid height less than 0.015 m and a potential energy below 5 mJ (Fig. 7c). However, if a vial with a fluid height of less than 0.015 m was combined with a higher potential energy (greater than 5 mJ), which corresponds to higher drop heights, cavitation would occur. Conversely, if the fluid height was greater than 0.015 m, cavitation was present at lower potential energies. When observing the difference between cavitating and non-cavitating behavior as compared to base cross-sectional area versus potential energy, vials with a base cross-sectional area less than 1.5e-04 m^2^ cavitate, while vials with a larger base cross-sectional area (above 2.5e-04 m^2^) cavitate at greater potential energies (above 6 mJ) (Fig. 7d). Given that there was not a clear/shared standard between glass and plastic vials to determine the onset of cavitation, we decided to look at another metric; base area/fluid height versus potential energy (Fig. 8). Using this metric, a trend was found showing that cavitation never occurred for a base area/fluid height greater than 0.01 m, and for a potential energy less than 5.3 mJ.

### Fluid type

When comparing buffer and DI water between similar drop heights, vial fill and material, as well as cavitation occurrence, this creates 28 different conditions to compare between fluid types. Of those 28 cases, 16 were dominated by buffer; 10 of which the vial material was glass. For buffer in glass vials, buffer had a higher occurrence of no cavitation (6 total) across all drop conditions when compared to water in glass vials (4 total). Additionally, both fluids in glass vials had the same number of occurrences of cavitation (4 each) across the fill volumes and drop heights. When comparing plastic vials, they performed in almost the same manner with buffer having a slight lead in presence of cavitation as compared to DI water (3 vs 2 respectively) across drop heights. Both buffer and DI water have 3 cases of no cavitation occurring in plastic vials. Buffer and DI water had the same number of occurrences (4) of no cavitation observed in glass and plastic vials. The drop and fill conditions were 1 ml filled glass vials dropped from a height of 0.2 m, 0.8 m, and 1.0 m and 2 ml filled plastic vials dropped from a height of 0.2 m. For a 2 ml filled plastic vial dropped from a height of 0.2 m, both buffer and water had one occurrence of observed cavitation each.

When ignoring drop conditions and vial material, there were 107 total buffer filled vials dropped, and 101 DI water filled vials (Table 3). Of the 107 buffer vials, 61 (57%) drops had no cavitation. A total of 24 (39.3%) of those 61 no cavitation drops occurred on the first drop, while the remaining 37 vials (60.7%) showed no cavitation on subsequent drops. Of the 46 buffer vials that cavitated, 14 (30.4%) cavitated on the first drop, while 32 (69.6%) cavitated on subsequent drops. Of the 101 DI water vials, 51 (50.5%) had no cavitation. Of the 51 vials that did not experience cavitation, 21 (41.2%) did not cavitate on the first drops, while the remaining 30 vials (58.8%) did not cavitate on subsequent drops. Of the 50 DI water vials that cavitated, 15 (30%) cavitated on the first drop, and 35 (70%) cavitated on subsequent drops.

**Table 3:** Data of all drops based upon fluid type.

	Water	Buffer	Water (vials that sat all night removed)	Buffer (vials that sat all night removed)
*Total drops*	101	107	68	71
#of no cavitation drops	51 (50.5%)	61 (57%)	34 (50%)	37 (52.1%)
#of cavitation drops	50 (49.5%)	46 (43%)	34 (50%)	34 (47.9%)
No cavitation 1^st^ drop	21 (41.2%)	24 (39.3%)	14 (41.2%)	16 (43.2%)
Cavitation 1^st^ drop	15 (30%)	14 (30.4%)	8 (23.5%)	7 (20.6%)
No cavitation on 2^nd^ + drops	30 (58.8%)	37 (60.7%)	20 (58.8%)	21 (56.8%)
Cavitation on 2^nd^ + drops	35 (70%)	32 (69.6%)	26 (76.5%)	27 (79.4%)
*Total drops counted*	208		139	

### Rest period between drops

Vials that sat overnight did not cavitate on their first drop but did on a second drop. 69 vials sat overnight before being dropped; of which there was 33 DI water filled vials and 36 buffer filled vials (Table 4). Of the 33 DI water vials, 18 (54.5%) had no cavitation. Of the 15 drops that cavitated, 8 (53.3%) cavitated on the first drop, while the remaining 7 (46.7%) cavitated on a second drop. Of the 36 buffer filled vials, 23 (63.9%) had no cavitation. Of the 13 that cavitated, 7 (53.8%) cavitated on the first drop, while the remaining 6 (46.2%) cavitated on a second drop.

**Table 4:** Data of drops that sat all night.

	Water	Buffer
*Total drops*	33	36
#of no cavitation drops	18 (54.5%)	23 (63.9%)
#of cavitation drops	15 (45.5%)	13 (36.1%)
No cavitation on 1^st^ drop	8 (44.4%)	8 (34.8%)
Cavitation on 1^st^ drop	8 (53.3%)	7 (53.8%)
No cavitation on 2^nd^ + drops	10 (55.6%)	15 (65.2%)
Cavitation on 2^nd^ + drops	7 (46.7%)	6 (46.2%)
*Total drops that sat all night*	69	

**Figure 9:**
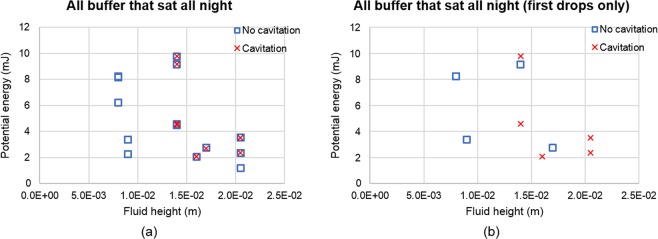
Fluid height vs. Potential energy for buffer solutions that sat all night. Left graph has all buffer data that sat all night. Right graph only shows the first drop of buffer filled vials that sat all night.

When looking at how rest period may affect cavitation behavior, Figures SD1a to SD1h (Supplementary Dataset) did not provide a shared standard at which to determine the onset of cavitation. However, fluid height provided a common standard when looking at rest period effects on buffer solution (Fig. 9); when the fluid height is less than 0.01 m, cavitation will not occur.

### Vial material

There is a significant difference between plastic and glass vials for the onset of cavitation. A total of 209 drops were analyzed, from which 124 (59.3%) were glass vials and 85 (40.7%) were plastic (Table 5). Of the 124 glass vials that were dropped, 46 (37.1%) had cavitation. Of the 78 no cavitation cases, 31 (39.7%) had no cavitation on the first drop, while the remaining 60.3% had no cavitation on the subsequent drops. Of the 46 drops with cavitation 30.4% cavitated on the first drop, and the remaining 69.6% cavitated on subsequent drops. Of the 84 plastic vials that were dropped, 49 (58.3%) cavitated. Of the 35 no cavitation cases, 14 (40%) had no cavitation on the first drop, the remaining 21 (60%) had no cavitation on subsequent drops. Of the 49 cavitation cases, 15 (30.6%) had cavitation on the first drop, and the remaining 69.4% cavitated on subsequent drops.

**Table 5:** Data of drops based upon vial material.

	Glass	Plastic	Glass (vials that sat all night removed)	Plastic (vials that sat all night removed)
*Total drops by vial material*	124	84	97	42
# of no cavitation drops	78 (62.9%)	35 (41.7%)	59 (60.8%)	12 (28.6%)
# of cavitation drops	46 (37.1%)	49 (58.3%)	38 (39.2%)	30 (71.4%)
No cavitation 1^st^ drop	31 (39.7%)	14 (40%)	26 (44.1%)	4 (33.3%)
Cavitation 1^st^ drop	14 (30.4%)	15 (30.6%)	11 (28.9%)	4 (13.3%)
No cavitation on 2^nd^ + drops	47 (60.3%)	21 (60%)	33 (55.9%)	8 (66.7%)
Cavitation on 2^nd^ + drops	32 (69.6%)	34 (69.4%)	27 (71.1%)	26 (86.7%)
*Total Drops Counted*	208		139	

### Bottom geometry

**Figure 10:**
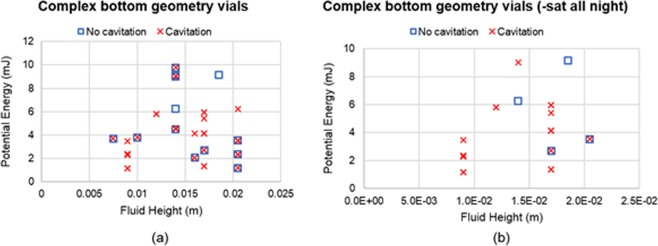
Fluid height vs potential energy for complex bottom geometry. (**a**) All data for complex geometry; (**b**) drops that sat all night removed.

**Figure 11:**
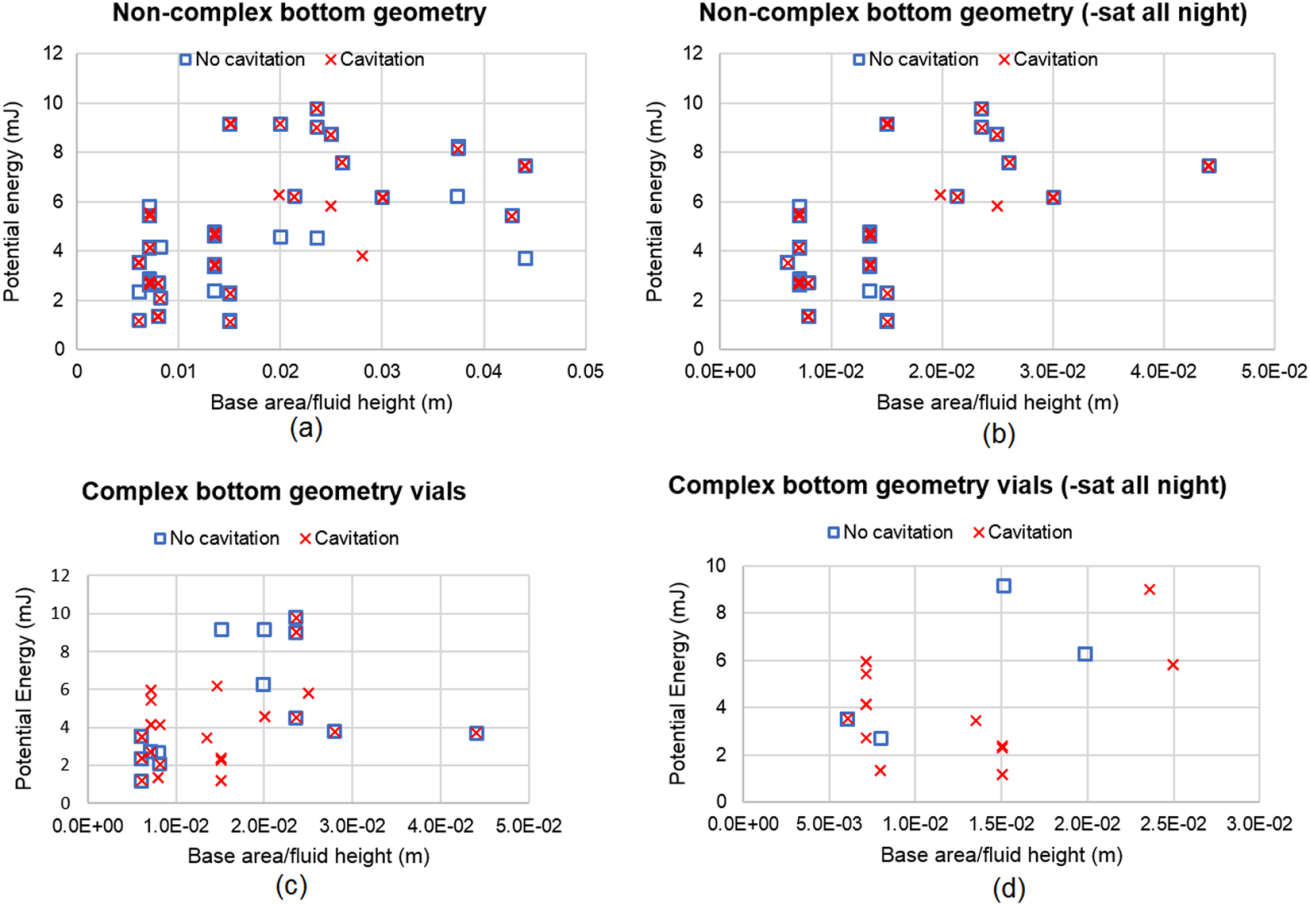
Base area/fluid height vs potential energy for non-complex bottom geometry for (**a**) all the data; (**b**) drops that sat all night removed; Base area/fluid height vs potential energy for complex bottom geometry for (**c**) all the data; (**d**) drops that sat all night removed. The results include both DI water and L-histidine buffer solutions.

When looking at how bottom geometry may affect cavitation behavior, Figures SD2a to SD2f (Supplementary Dataset) did not provide a clear standard at which to determine the onset of cavitation under the conditions we tested. However, fluid height provided a standard when looking at complex geometry where drops that sat all night were removed from the graph (Fig. 10b). Given that there was not a shared standard between non-complex and complex bottom geometry vials to determine the onset of cavitation, we decided to look at base area/fluid height versus potential energy separately (Fig. 11a,b). Using this metric, a trend was found for complex bottom geometry with drops that sat all night removed, showing that cavitation never occurred for a base area/fluid height greater than 0.01 m, and for a potential energy less than 6mJ. This metric, however, did not provide a trend for the non-complex geometries.

## Discussion

If a patient or caregiver inadvertently drops a vial and it impacts a hard surface, the resulting shock has been shown to have the potential to initiate cavitation at drop heights as low as 20 cm. As the drops are unintentional, their impact conditions (including impact angle) are often uncontrollable and highly variable. Our results support the idea that impact angle has an influence on the rate of cavitation. Figure 6 showed that for side impacts (angles less than 30°) no cavitation occurred. However, for impact angles greater than 30° there was no significant difference between the percentage of vials that cavitate versus those that do not. The impact concentrated in a smaller region of the vial (angles greater than 30°) induces a pressure wave with greater intensity in the vial. This causes cavitation as the high-intensity pressure wave returns to normal pressure.

Previous studies^5,9,24^ suggested that the fill volume and the amount of headspace in vials play a role in not only cavitation phenomena but also protein stability and its potential aggregation. Randolph *et al*.^5^ observed that the fill volume of vials matters, but these observations were made using a different vial drop methodology. Aiming to study fill volume, Randolph *et al*.^5^ carried out an experiment in which vials were dropped using the Lansmont tower from a height of 40 inches while filled with either 1 mL or 3 mL of mAb1 solution. The 3 mL filled vials had a more extensive cavitation phenomena than compared to vials filled with 1 mL of mAbl solution. This also held true for vials filled with either 1 mL or 4 mL of 1.75 mg/mL rhGH which were also dropped from a height of 40 inches. They found that prior to dropping, the vials containing rhGH had an average of 11,100 particles of size equal to or greater than 2 µm/mL. After dropping, the particle concentration of the 1 mL rhGH filled vials increased to 16,800 ± 700 particles/mL, and the 4 mL rhGH filled vials increased to 31,300 ± 3500 particles/mL. Regarding drop height, during their FlowCAM analysis they found that drop shock increased the number of particles overall, but that drop height did not appear to have a significant effect on the number of particles counted; the particle counts for all drop heights were roughly equal. This agrees with both our findings, and the findings of Torisu *et al*.^9^, that a higher fill volume results in an increase in cavitation phenomena as compared to lower fill volumes. More importantly, we found agreement between our findings and Randolph’s findings even using different drop methods (i.e. free hand and tower).

Using a larger volume vial (larger diameter) to reduce fill volume works too. Taller vials are not desirable because of possible drawbacks related to the need for longer needles. Based on the results, cavitation can be reduced by lowering the impact energy per base area, meaning that there needs to be an increase in vial diameter, which leads to a decrease in the fluid height. A 5 ml vial filled with 1 ml of solution has a larger base area and lower fluid height when compared to a 2 ml vial filled with 1 ml of solution. Comparing those two vials, the 5 ml vial had a lower probability of cavitation occurrence. Additionally, cavitation never occurred in glass vials where the fluid height was less than 10 mm, meaning that the ratio of base area/fluid height has the potential to mitigate cavitation depending on the drop height. As the fluid height will decrease with time as the patient consumes the drug, using vials with a larger base area could be a possible strategy to mitigate cavitation caused by drop shock. Kiese *et al*.^24^ also investigated the effects of fill volume on aggregation, finding that shaking the IgG1 solution with headspace induced both visible particles and soluble aggregates. Conversely, samples without headspace did not show any significant amounts of subvisible particles after being stressed. However, our research did not use the same particle detection methods that Kiese *et al*.^24^ did, nor did they look at vials undergoing drop shock. Therefore, while we may have noticed a decrease of cavitation phenomena when paired with decreased fill volume, our samples may have been subjected to increased aggregation due to the renewal of the air-solution interface.

There was no significant difference in the behavior of cavitation between DI water and the buffer solution; 49.5% of the DI water filled vials and 43% of the buffer vials had cavitation. The lack of significant difference is believed to be attributed to the fluid properties; both the density and viscosity have a percent difference of less than 3%. Additionally, there was no observable difference in the characterization of cavitation between water and buffer solutions. The 10mM L-histidine buffer solution and DI water have similar densities at 999.761 kg/m^3^ and 974.067 kg/m^3^ respectively, resulting in a 2.6% difference. The buffer solution is a more viscous fluid than DI water with respective viscosities of 0.8899*10^−3^ kg/m.s and 0.89*10^−3^ kg/m.s, resulting in a difference. The surface tension of DI water is 72.86 mN/m, while the surface tension of the buffer solution was not predetermined but is believed to be like water.

When comparing the data between vials that did not sit all night (Table 3) before being dropped and vials that did (Table 4), there is a slight decrease in the percentage of vials that did experience cavitation. Vials that sat overnight experienced a 4.5% and 11.8% decrease in the occurrence of cavitation for both DI water filled vials and buffer filled vials, respectively. One hypothesis for the decrease in cavitation after the vials sitting overnight is that there may be a reduction in the amount of dissolved gases in the bulk fluid, therefore reducing the available nucleation sites for cavitation. This theory is supported by Li *et al*.^26^ whose experimental results indicate that an increase in the amount of dissolved gases causes an increase in acoustic cavitation due to a decrease in the tensile strength of the fluid.

When looking at the overall cavitation behavior of material, regardless of drop conditions and fluid type, the plastic vials had a higher percent of cavitation (58.3%) as compared to the glass vials (37.1%). A possibility to explain the increase in cavitation behavior for the plastic vials may be attributed to the complex bottom geometry that is present in the plastic vials. The inflection points allow for more concentrated low-pressure regions after impact, thus potentially providing more nucleation sites. However, Sederstrom^27^ performed CFD simulations of two different vial bottom geometries; flat and simple convex, where the vials were simulated to drop from the average height of an individual’s hand to the floor and with 0.5” of padding. The localized low pressure (60.8 kPa) at the bottom of the vials did not change as a function of shape, supporting the idea that cavitation is not likely to occur for the flat and convex bottoms if padded. It is possible that without the padding, the simulations may have shown that the low pressure may change as a function of shape. Additionally, Sederstrom^27^ simulated the difference between glass and plastic vials, finding that plastic has a higher low-pressure value which results in a weaker pressure wave. The weaker pressure wave is to be expected due to plastic having a much smaller Young’s Modulus and greater ability to deform, and thus having less chance to cavitate. However, our results disagree as we observed plastic vials having more cavitation cases then the glass vials.

Maruno *et al*.^28^ experimentally investigated how surface properties of prefilled syringe barrels can affect the level of micron aggregates and protein adsorption. The referred study by Maruno *et al*.^28^ found that the level of protein adsorption for glass syringes is higher than the level of adsorption for plastic syringes. Even though these results contradict our findings concerning influence of vial material on the onset of cavitation, the solutions in the study carried out by Maruno *et al*.^28^ were under mechanical stress from the aspiration and ejection sampling methods. In our current study, the glass vials had less cavitation cases because glass surfaces partially absorb energy from the impact of the vial with the floor. Consequently, the vial itself experiences the greatest amount of mechanical stress, reducing the intensity of the localized spots in the fluid with gradients of pressure and temperature. The pressure wave originated from the impact has a lower intensity as compared to the plastic vials. Plastic surfaces absorb much less energy from the impact when the vial strikes a hard surface (e.g. floor), resulting in a more intense pressure wave. Torisu *et al*.^29^ carried out an experiment in which glass prefilled syringes were subjected to a combination of mechanical stresses from shaking and dropping. In this study, the type of mechanical stresses exerted on the syringes (shaking and dropping) is similar to the one considered in our current study (only dropping). The referred study by Torisu *et al*.^29^ did not, however, compare plastic and glass syringes. There are no precedents of studies in literature that addressed differences between glass and plastic therapeutic vials under mechanical stress from hand-dropping.

When comparing the contact angle between the glass and plastic vials (5–65° and 91 ± 2°, respectively), glass has a smaller contact angle than the plastic vials, which means it has a greater wettability and is more hydrophilic. This in turn may mean that the glass vials have a greater probability of having protein adsorbed to the glass vial surface due to the increased liquid-solid interaction. Fang *et al*.^30^ found that Bovine Serum Albumin had a greater adsorption for clean type 1 borosilicate glass, then silicon oil treated glass which has a contact angle around 95–110°. Thus, the larger the contact angle of the plastic vials makes them more ideal than glass vials in terms of protein adsorption.

## Conclusions

Our results indicated that fluid fill height and vial base diameter play a large role in cavitation mitigation. Decreasing fill height by using a smaller fill volume or larger diameter vials were found to mitigate cavitation across drop heights. When comparing larger diameter vials (i.e. 5 mL) to smaller vials (i.e. 2 mL) filled with the same volume of solution, the larger diameter vial had a lower probability of cavitation occurrence. Our results also indicated that there was not a significant difference between the cavitation behavior of DI water and L-Histidine buffer solution. Lastly, there was a significant difference between the cavitation behavior between glass and plastic vials, and plastic had more cavitation cases.

Future studies should quantify how internal geometry may affect cavitation, and conditions at which cavitation occurs. Further work will need to be done by either creating unique bottom geometries in the glass vials, or through the removal of the inflexion points in the plastic vials. Additionally, a future work focusing on the investigation of wall thickness influence on cavitation could be greatly valuable to make progress on determining how cavitation is related to geometric aspects of the vials. Moreover, performing CFD simulations of the vials with complex bottom geometries may shed light into the low-pressure region, and how changing the complexity of the geometry may affect it. Furthermore, future experiments performing degassing can be highly valuable to consistently demonstrate that vials sitting overnight can have a positive impact on cavitation by decreasing its occurrence.

## Supplementary information

Supplementary Dataset 1

Cavitation Video Recording: Example
