# Supplementary material for: Parameters Influencing Cavitation Within Vials Subjected to Drop Shock
Source: Sci Rep. 2019 Dec 16;9:19210. doi: 10.1038/s41598-019-55668-9 (PMC6915723; doi:10.1038/s41598-019-55668-9)
Supplement: Supplementary file 1 — Supplementary Dataset 1 [file 41598_2019_55668_MOESM1_ESM.pdf]

RESEARCH ARTICLE

Parameters Influencing Cavitation Within Vials Subjected to Drop Shock

Rafael Valotta Rodrigues[1], Meagen Puryear[1], Donn Sederstrom[2], Corinne Lengsfeld[1]

Supplementary Dataset 1

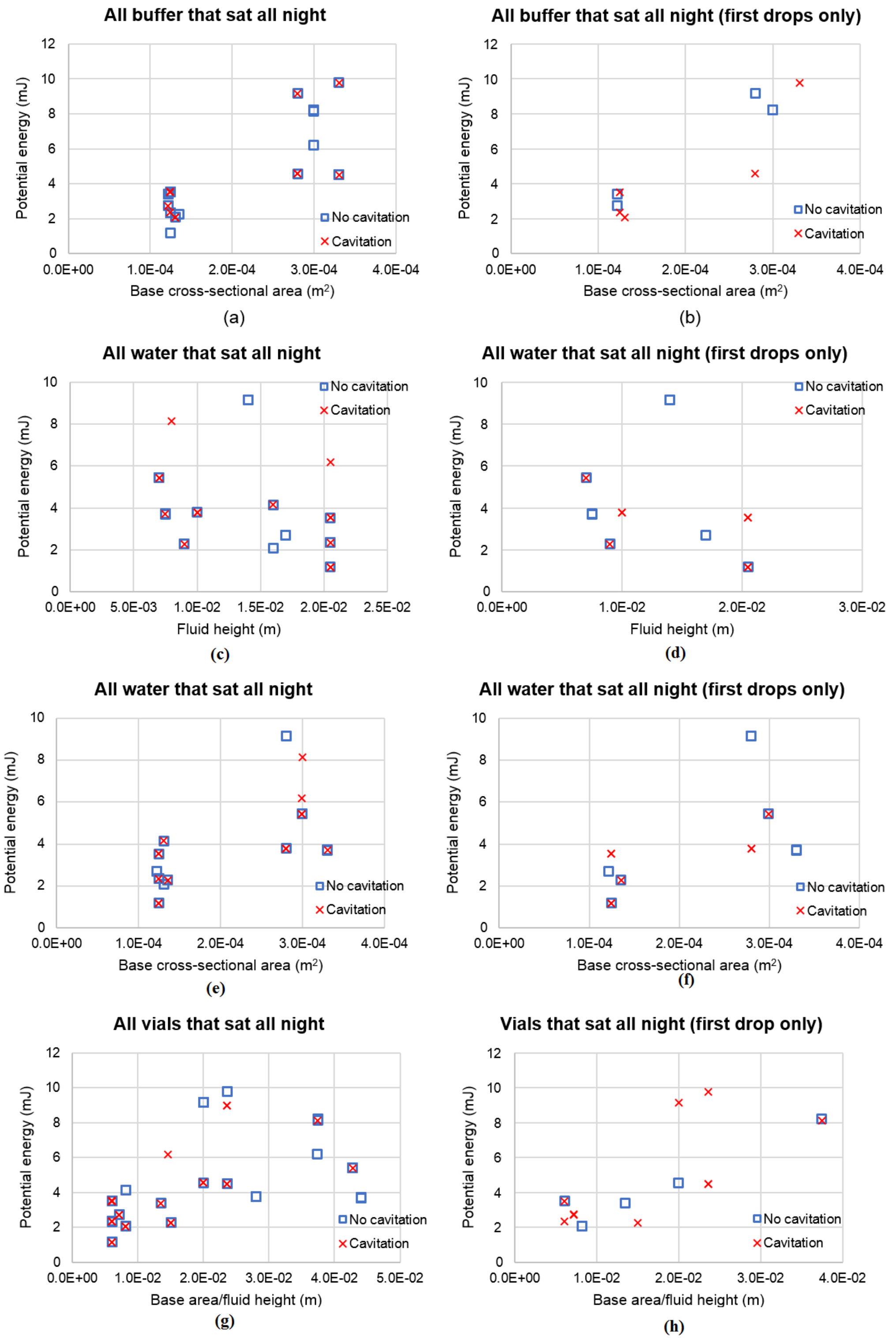

Figure SD1. Base cross-sectional area vs. Potential energy for Buffer solutions that sat all night for a) all buffer data that sat all night; b) only shows the first drop of buffer filled vials that sat all night. Fluid height vs. Potential energy for water solutions that sat all night for c) all water data that sat all night; d) only shows the first drop of water filled

vials that sat all night. Base cross-sectional area vs. Potential energy for water solutions that sat all night for e) all water data that sat all night; f) only shows the first drop of water filled vials that sat all night. Base area/fluid height vs potential energy for vials that sat overnight for g) all vials; h) only show the first drop of those that sat overnight.

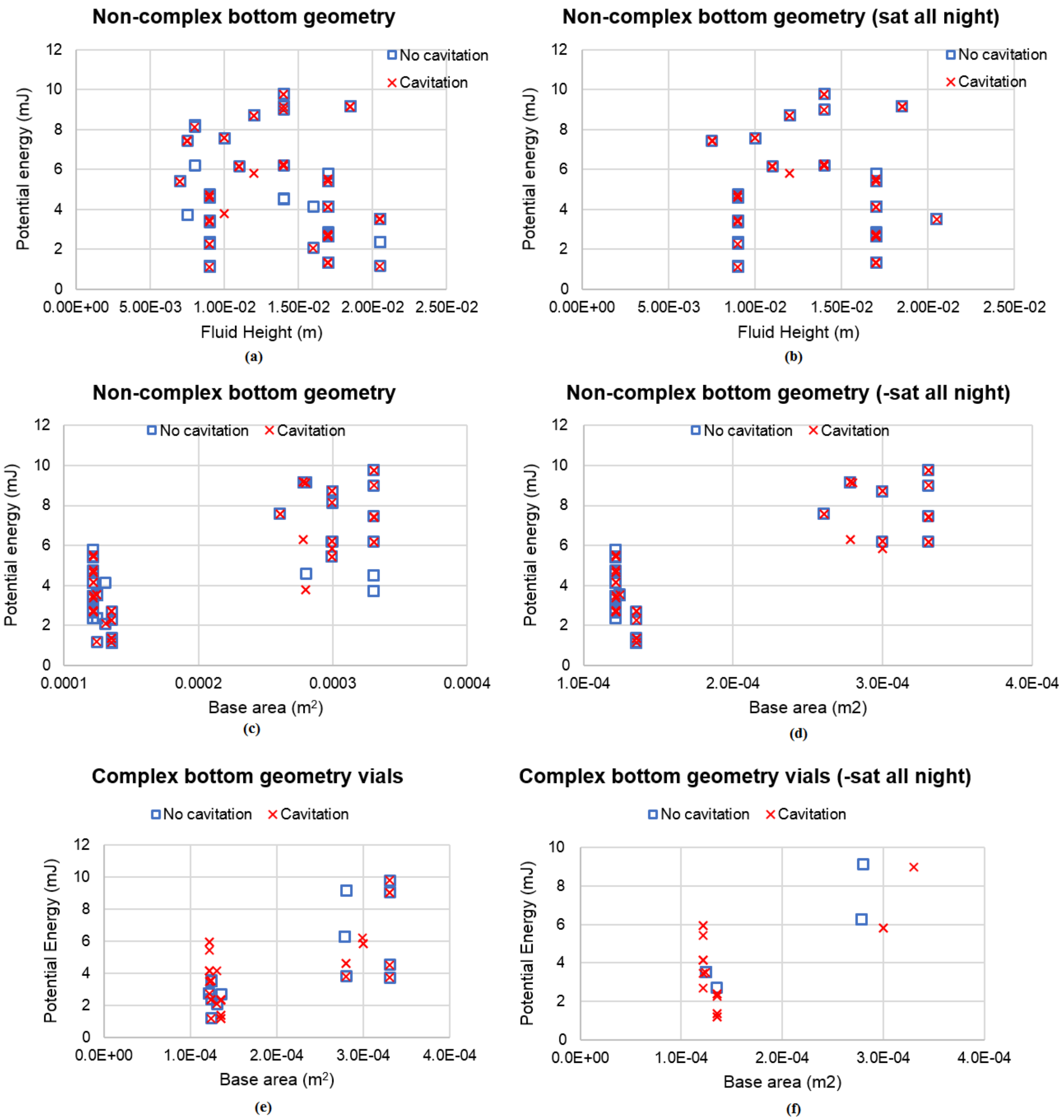

Figure SD2. Fluid height vs potential energy for non-complex bottom geometry for a) all data; b) drops that sat all night removed. Base cross-sectional area vs potential energy for non-complex bottom geometry for c) all data; d) drops that sat all night removed. Base cross-sectional area vs potential energy for complex bottom geometry for e) all data; f) drops that sat all night removed.
